# The Hippo-YAP Pathway Regulates 3D Organ Formation and Homeostasis

**DOI:** 10.3390/cancers10040122

**Published:** 2018-04-17

**Authors:** Erika Ishihara, Hiroshi Nishina

**Affiliations:** Department of Developmental and Regenerative Biology, Medical Research Institute, Tokyo Medical and Dental University (TMDU), 1-5-45 Yushima, Bunkyo-ku, Tokyo 113-8510, Japan; ishihara.dbio@mri.tmd.ac.jp

**Keywords:** YAP, Hippo, organogenesis, homeostasis, cell elimination, hepatocyte

## Abstract

The vertebrate body shape is formed by the specific sizes and shapes of its resident tissues and organs, whose alignments are essential for proper functioning. To maintain tissue and organ shape, and thereby function, it is necessary to remove senescent, transformed, and/or damaged cells, which impair function and can lead to tumorigenesis. However, the molecular mechanisms underlying three-dimensional (3D) organ formation and homeostasis are not fully clear. Yes-associated protein (YAP) is a transcriptional co-activator that is involved in organ size control and tumorigenesis. Recently, we reported that YAP is essential for proper 3D body shape through regulation of cell tension by using a unique medaka fish mutant, *hirame* (*hir*). In Madin–Darby canine kidney (MDCK) epithelial cells, active YAP-transformed cells are eliminated apically when surrounded by normal cells. Furthermore, in a mosaic mouse model, active YAP-expressing damaged hepatocytes undergo apoptosis and are eliminated from the liver. Thus, YAP functions in quantitative and quality control in organogenesis. In this review, we describe the various roles of YAP in vertebrates, including in the initiation of liver cancer.

## 1. Introduction

Despite the fact that living organisms are continually faced with the force of gravity, they are able to maintain complicated and elaborate three-dimensional (3D) organs [1]. The reason why living organisms do not collapse from their own weight is that they have a mechanism for gravitational resistance inside their own cells. This mechanism is known as cellular tension, which employs actomyosin-generated contraction to resist collapse. Actomyosin-mediated cellular tension constantly affects the extracellular matrix (ECM) and adjacent cells, and influences mechanohomeostasis [2,3]. In recent years, it has become clear that mechanohomeostasis is fundamental to cellular and tissue health [4]. Mechanohomeostasis is closely involved in the regulation of cell proliferation, differentiation, and apoptosis, and the breakdown of this mechanism leads to various pathologies such as cancer and arteriosclerosis caused by chronic inflammation [5,6].

Tissues and organs undergo cellular stress that can lead to damaged, senescent, and/or transformed cells [7,8,9,10] that require elimination. The loss of these cells must then be compensated for by cell proliferation, which maintains the size and quality of the tissues and organs [11,12,13,14]. It has been reported that oncogene-induced senescent hepatocytes secrete chemo- and cytokines and are subject to immune-mediated clearance (designated as “senescence surveillance”), which depends on an intact CD4+ T-cell-mediated adaptive immune response [15]. Impaired immune surveillance of pre-malignant senescent hepatocytes results in the development of murine hepatocellular carcinomas (HCCs), thus showing that senescence surveillance is important for tumor suppression in vivo. “Cell competition” is a type of cell–cell interaction that was originally discovered in the imaginal wing disc of *Drosophila* [16]. During cell competition, the fitness of a cell is compared to that of its neighboring cells. Cells that are less fit than their neighboring cells are “losers” and are eliminated by either apoptosis or apical extrusion, while the fitter cells become “winners” and survive [17,18]. For example, in the *Drosophila* wing disc, the ribosomal proteins-encoded *Minute* gene heterozygous cells underwent apoptosis as losers when they were confronted with wild-type (WT) cells [19,20]. And, in mouse embryonic stem cells or epiblasts, the cells with lower levels of Myc underwent apoptosis and were eliminated [21,22]. Finally, in Madin–Darby canine kidney (MDCK) epithelial cells, those cells that expressed the oncogenic proteins v-Src or K-Ras (G12V) were losers and became apically extruded [23,24].

Yes-associated protein (YAP) and its paralog, transcriptional co-activator with PDZ-binding motif (TAZ) are well-known downstream effectors of the Hippo signaling pathway, which is an evolutionarily conserved regulator of organ size control during animal development, tumorigenesis, regeneration, and stem cell self-renewal [25,26]. The Hippo signaling pathway negatively regulates YAP by phosphorylating five conserved serine residues. Phosphorylated YAP binds to the phosphoserine/phosphothreonine-binding protein 14-3-3, which retains YAP in the cytoplasm leading to its degradation [27,28]. Inactivation of the Hippo pathway enables YAP translocation into the nucleus where it drives target gene expression through binding to various transcription factors, including TEA domain transcription factor (TEAD)1/2/3/4, Smad1/2/3, p73, Kruppel-like factor 5 (KLF5), Runx1/2, ErbB4, T-box transcription factor 5 (TBX5), and FoxO1 [29,30,31]. YAP and TAZ regulate various cellular responses such as cell proliferation, apoptosis, contact inhibition, epithelial-mesenchymal transition, and cell competition [25,26].

In this review, we describe our recent reports that the Hippo-YAP pathway is involved in 3D organ formation and abnormal cell elimination in vivo. We first outline the molecular basis of YAP-regulated cellular tension and organ formation, which functions via the cortical actomyosin network. In the latter part, we describe the molecular mechanisms of YAP-induced cell elimination in vitro and in vivo, which occurs via cytoskeleton organization and cell migration and Rho-family GEFs. Finally, we discuss the role of these processes in liver cancer, and in cancer in general. These apparently diverse aspects of YAP function are essential for cell, tissue, and body homeostasis. Thus, the loss of these functions induces diseases including cancer.

## 2. YAP Regulates 3D Organ Formation through Cell Tension in Medaka

**Figure 1:**
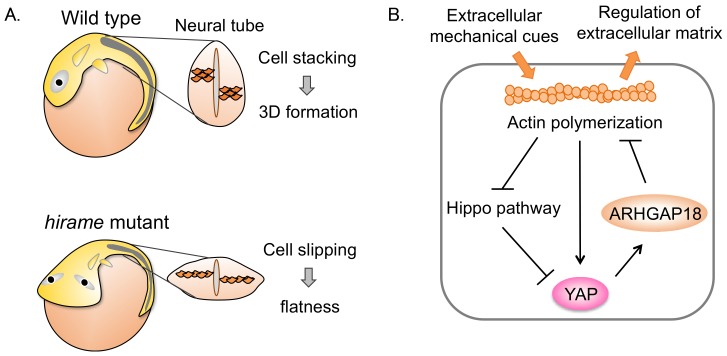
Yes-associated protein (YAP) regulates mechanohomeostasis in medaka. (**A**) Wild-type (WT) medaka embryo and *hirame* mutant. *hirame* mutant displays a flat body due to cell slipping. (**B**) A model of mechanohomeostasis. Extracellular mechanical cues induce F-actin polymerization and activate YAP. Activated-YAP activates Rho-GTPase activating-protein 18 (ARHGAP18) and suppresses F-actin polymerization, resulting in regulation of the extracellular matrix. Thus, YAP, the cytoskeleton and the extracellular matrix constitute a feedback loop.

To identify new factors related to organ formation, we analyzed a large-scale mutagenesis screen using medaka [32]. From this, we identified the medaka *hir* mutant, which displays a flattened body and exhibits progressive body collapse (Figure 1A). This was associated with flattening and misalignment of tissues and organs, including the neural tube, somites, and lenses. Positional cloning in *hir* mutants identified a nonsense mutation in the YAP gene, and morpholino oligonucleotides showed that knockdown of YAP in medaka embryos phenocopied this *hir* phenotype [33,34]. We further evaluated the contribution of TAZ to the YAP knockdown phenotype. YAP/TAZ double knockdown showed more obvious blastopore closure than the YAP knockdown alone. In *hir* embryos, cell proliferation was largely unaffected, but it was strongly reduced in the TAZ knockdown and YAP/TAZ double knockdown medaka embryos.

To examine whether body collapse correlated with the direction of gravity, mutant embryos were maintained either right-side or left-side down. Mutant embryos collapsed towards the earth. Thus, *hir* embryos could not withstand external forces, suggesting reduced tissue tension. Indeed, micropipette aspiration experiments indicated that the physical tissue tension of the neural tube in *hir* was reduced. Single-cell tracking analysis revealed that tissue flatting was associated with a failure to stack cells, and an increase in cells slipping to one side after perpendicular cell division. In addition, *hir* retina showed punctate fibronectin patches. Thus, we concluded that YAP is required for actomyosin-mediated tissue tension and functions in tissue alignment by regulating fibronectin assembly in medaka.

We used a human 3D spheroid in vitro culture system to identify the downstream effectors of YAP that regulate tissue tension. YAP knockdown spheroids collapsed when they were exposed to forces applied by slow centrifugation. YAP knockdown spheroids also showed reduced levels of actomyosin activity, specifically reduced levels of phosphorylated myosin regulatory light chain (pMRLC), and also lacked the typical pattern formation of fibronectin fibrils. YAP knockdown spheroids were also subjected to gene expression profiling, which revealed significantly altered expression levels of the Rho GTPase activating protein (GAP), Activated-YAP activates Rho-GTPase activating-protein 18 (ARHGAP18). ARHGAP18 inhibits the small GTP-binding protein Rho thereby suppressing F-actin polymerization. The expression levels of ARHGAP18 were reduced in both the *hir* mutant and YAP knockdown spheroids. In ARHGAP18 knockdown spheroids, pMRLC levels were also reduced and fibronectin assembly was induced, similar to the phenotype in the YAP knockdown spheroids. Thus, these results suggest that YAP activates the actomyosin network and induces fibronectin assembly through regulating the expression of ARHGAP18.

We concluded that YAP plays a critical role in tissue tension via ARHGAP18 and associated genes by regulating the formation of the cortical actomyosin network, and that this mechanism is essential for producing the correct shape of the organ/body. Previous reports had shown that YAP can act as a mechanosensor in response to extracellular forces [35]. Our data indicate that YAP also acts as a mechanoregulator of tissue tension. Extracellular mechanical cues induce YAP activation through actin polymerization. Then, YAP regulates ARHGAP18 activity, resulting in actin-dependent extracellular matrix formation. ARHGAP18 might suppress YAP activity through a negative feedback mechanism (Figure 1B). This implies mechanohomeostasis: a feedback loop where YAP controls tissue tension and, in turn, is controlled by tissue tension.

## 3. YAP-Expressing MDCK Cells Undergo Apical Extrusion Depending on Neighboring Cell Status

**Figure 2:**
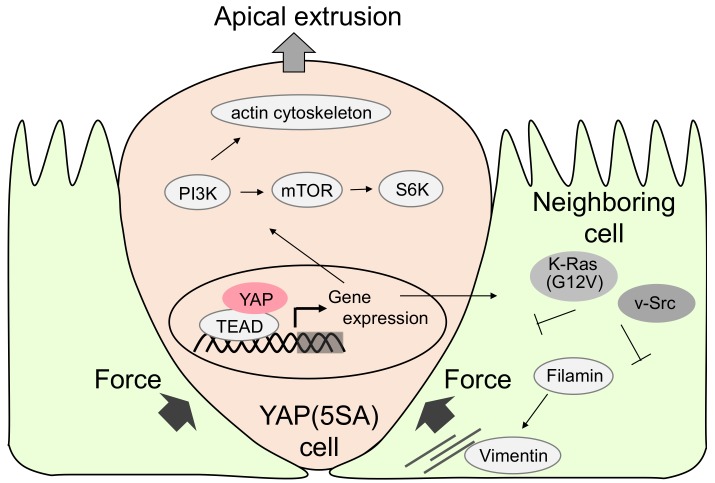
Molecular mechanism of apical extrusion of active YAP-expressing Madin–Darby canine kidney (MDCK) cells. Active YAP induces TEA domain transcription factor (TEAD)-dependent gene expression, which activates Phosphoinositide 3-kinase (PI3K) and Mammalian target of rapamycin (mTOR) signals. This is recognized by neighboring cells in which filamin accumulation induces pressure for apical extrusion of YAP active cells. In contrast, active Ras or Src in the neighboring cell inhibits apical extrusion.

The elimination of transformed cells is one of the functions required for organ homeostasis [36]. Previous reports showed that K-Ras (G12V) or v-Src-expressing MDCK cells are extruded apically [23,24]. To investigate the fate of active YAP-expressing MDCK cells, we established YAP (5SA)-expressing MDCK cell lines (YAP (5SA) cells) that express constitutively active YAP (the five phosphorylatable serine residues were replaced by alanine residues). As a control, we used YAP wild-type (WT)-expressing MDCK cells (YAP (WT) cells). YAP (5SA) and YAP (WT) cells were labeled with a red fluorescent dye and were mixed with normal MDCK cells at a ratio of 1:50 (to represent a mosaic condition). Cell proliferation and cell survival were not different among YAP (5SA), YAP (WT), and normal MDCK cells. However, YAP (5SA) cells, but not YAP (WT) cells, were extruded apically when surrounded by normal MDCK cells (Figure 2). On the other hand, normal MDCK cells were not extruded when they were surrounded by YAP (5SA) cells. These results indicated that YAP activation induces apical extrusion in mammalian epithelial cells.

To identify the molecular mechanisms of YAP-induced apical extrusion, we analyzed the effects of YAP (5SA) domain mutants and specific chemical inhibitors on apical extrusion. YAP (5SA/TEAD*) lacking the TEAD-binding domain, and YAP (5SA/ΔPDZ) lacking the PDZ binding motif did not induce apical extrusion. The application of chemical inhibitors of phosphoinositide-3-kinase (PI3K), mammalian target of rapamycin (mTOR), or p70S6 kinase (p70S6K) also did not induce apical extrusion. These results indicated that YAP and TEAD-dependent gene expression, and the PI3K-mTOR-S6K pathway, are essential for apical extrusion. Previous reports showed that vimentin, an intermediate filament protein, and filamin, a homodimeric actin-binding protein, in neighboring MDCK cells are important for K-Ras (G12V)- and v-Src-inducing apical extrusion. To evaluate the importance of vimentin and filamin in neighboring MDCK cells, we used vimentin shRNA or filamin shRNA. Neighboring vimentin or filamin-knockdown MDCK cells inhibited the apical extrusion of YAP (5SA) cells. These data indicated that vimentin and filamin in neighboring cells regulate apical extrusion.

To investigate whether the condition of the neighboring cells affects the fate of YAP (5SA) cells, we compared apical extrusion-inducing activity between YAP (5SA), K-Ras (G12V), and v-Src cells. The apical extrusion of YAP (5SA) cells surrounded by K-Ras (G12V) or v-Src cells was greatly suppressed compared to co-culturing with normal MDCK cells. The apical extrusion of K-Ras (G12V) cells surrounded by v-Src was also completely suppressed. In contrast, apical extrusion of v-Src cells surrounded by K-Ras (G12V) or YAP (5SA) cells was not suppressed. Thus, normal MDCK cells have a stronger extruding activity than YAP (5SA), K-Ras (G12V), and v-Src cells. This demonstrates that, depending on the status of the neighboring cells, YAP (5SA) cells can change their fate with regards to apical extrusion.

## 4. YAP Induces Damaged Hepatocyte Elimination Dependent on the Status of Liver Sinusoidal Endothelial Cells (LSECs)

To investigate the role of YAP in organ homeostasis in vivo, we examined the dynamics of YAP (5SA) cells in a mosaic condition in the mouse liver. We utilized adenoviral infection and hydrodynamic tail vein injection (HTVi) to produce a ∼30% mosaic state [37]. YAP (5SA)-expressing hepatocytes generated by adenoviral infection proliferated. In contrast, YAP (5SA)-expressing hepatocytes were largely eliminated (to ∼3%) within seven days. YAP-activated hepatocytes in double knockout mice of the Hippo pathway components Mst1/Mst2 or Mob1a/Mob1b, prepared by HTVi, were also eliminated [38,39,40]. Importantly, in immune-deficient mice lacking T cells, B cells, and NK cells, YAP (5SA)-expressing hepatocytes were eliminated, which suggests that active YAP-expressing hepatocytes damaged by HTVi are eliminated independently of adaptive immunity.

**Figure 3:**
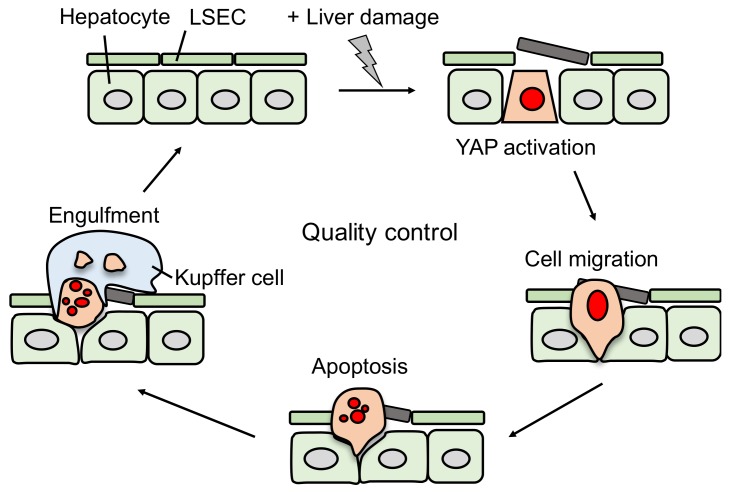
Model of YAP-induced damaged hepatocyte elimination. Hepatocytes expressing activated YAP in the presence of liver injury such as ethanol migrate into sinusoids, undergo apoptosis, and are engulfed by Kupffer cells.

To uncover the mechanisms involved, we analyzed mouse liver sections stained with different markers including the macrophage marker F4/80, a liver sinusoidal endothelial cell (LSEC) marker Stab2, and another LSEC marker, LYVE1. Immunostaining showed that YAP (5SA)-expressing hepatocytes migrated to the hepatic sinusoids where they were engulfed by liver resident macrophages known as Kupffer cells. The depletion of Kupffer cells from the YAP-expressing mice with clodronate liposomes [41] suppressed the elimination of YAP (5SA)-expressing hepatocytes from the liver and increased the presence of TUNEL+ apoptotic cells. These results indicate that active YAP-expressing hepatocytes migrate to the hepatic sinusoids where they undergo apoptosis and are subsequently engulfed by Kupffer cells (Figure 3).

To investigate whether only specific types of liver injury induce hepatocyte elimination in YAP (5SA) mice, we treated them with either carbon tetrachloride (CCl_4_), which causes specific injury to hepatocytes; monocrotaline, which mainly causes LSEC injury; or ethanol, which damages both LSECs and hepatocytes. Loss of YAP (5SA)-expressing hepatocytes was only detected in livers of mice treated with ethanol, but not CCl_4_ or monocrotaline. These results indicated that damage to both LSECs and hepatocytes is required for hepatocyte elimination.

To identify the molecular mechanism underlying YAP-mediated elimination of damaged hepatocytes, we performed gene expression profiling and hierarchical cluster analyses. Gene ontology analysis identified CDC42, which is a small GTP-binding protein that regulates cytoskeleton organization and cell migration. Consistent with an active role, dominant-negative mutants of CDC42 or Rac suppressed the elimination of YAP-activated hepatocytes. cDNA microarray analysis identified Ect2 and Fgd3, which are guanine nucleotide exchange factors (GEFs) for CDC42 and Rac, respectively [42,43,44]. Both *Ect2* and *Fgd3* mRNAs were induced in YAP-activated damaged hepatocytes treated with ethanol, but not with CCl_4_. These results indicate that: (1) active YAP and TEAD induce *Ect2* and *Fgd3* mRNA; (2) Ect2 and Fgd3 activate CDC42 and Rac, and (3) Cdc42 and Rac regulate cytoskeleton organization and stimulate cell migration. We propose that YAP functions in an emergency stress response that eliminates damaged cells to maintain tissue homeostasis by cytoskeletal remodeling through Rho family GEFs.

## 5. YAP Activation and Cancer

Previous reports have shown that the Hippo-YAP pathway controls organ size and hepatocellular carcinogenesis: YAP overexpression in mouse liver induced hepatomegaly and hepatocellular carcinoma (HCC) [45,46]. In addition, liver-specific Mst1/2 deficient mice showed hepatomegaly and HCC formation through YAP activation [40]. Liver-specific Mob1a/1b double deficient mice also showed HCC formation [39]. Thus, YAP functions as an oncogene, promoting liver overgrowth and liver cancer formation.

**Figure 4:**
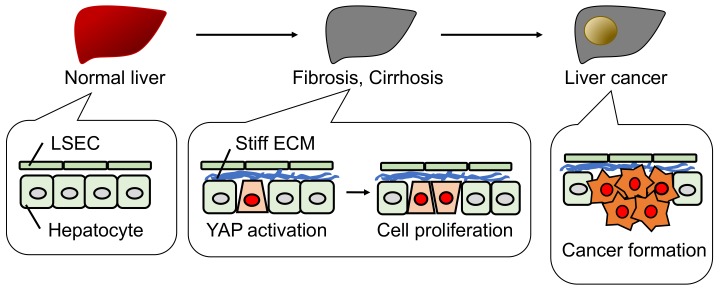
Schematic model of liver cancer formation. In fibrosis and cirrhosis, a stiff ECM activates YAP and promotes hepatocyte proliferation, but not hepatocyte elimination as shown in Figure 3. This pathological situation can lead to liver cancer formation.

We found a novel function for YAP in a tumor-suppressive role, as described above. We propose that elimination of YAP-induced damaged hepatocytes suppresses cancer formation and maintains liver quality (Figure 3). The damaged hepatocyte elimination is inhibited in fibrotic and cirrhotic livers due to a stiffened ECM containing collagen (Figure 4). Previous reports showed that a stiff ECM induces YAP activation via F-actin regulation [35]. In turn, YAP activation promotes hepatocyte proliferation, resulting in liver cancer formation [45,46]. Thus, in the presence of activated YAP, liver cancer is initiated dependent on the status of the surrounding environment, but not on Hippo pathway mutations. This mechanism might explain the fact that Hippo pathway mutations are extremely rare in human liver cancers [47].

Recent reports have shown that the Hippo-YAP pathway plays important roles in liver and kidney development [48,49]. Lats1/2 deletion in mouse liver results in perinatal lethality and failed to develop tumors. Similarly, Lats1/2 deletion in mouse kidney leads to loss of nephron formation, but not tumor formation. Another recent study has suggested that the Hippo-YAP pathway is involved in cancer immunity. A melanoma cell line lacking Hippo pathway components Lats1/2 was shown to induce anti-tumor immune responses in a syngeneic mouse model [50]. In this context, YAP nuclear translocation was enhanced and YAP target gene expression was increased. The melanoma cells secreted extracellular vesicles and induced a type I interferon response. Subsequently, the melanoma cells were destroyed by the host immune response. In contrast, liver-specific Mst1/2 or Lats1/2 knockout mice displayed an immunosuppressive microenvironment [51,52]. These knockout mice showed type II macrophages recruitment via cytokines CCL2 and CSF1 upon YAP activation. It remains unclear how these apparently opposing effects of Hippo component knockouts on promoting or repressing cancer immune surveillance can be reconciled [53].

The described diverse roles of YAP in cell tension, damaged cell elimination, and cancer immunity, are all essential for tissue formation and tissue homeostasis. Therefore, impaired YAP functions manifest as tissue overgrowth and cancer formation.

## 6. Conclusions

YAP knockout mice are embryonic lethal at embryonic day 8.5 and TAZ knockout mouse are viable with glomerulocystic kidney disease and pulmonary disease [54,55,56,57]. On the other hand, YAP knockout *hir* mutant showed the unique flatten phenotype. Our data using medaka has helped to define the separate roles for YAP and TAZ: TAZ regulates cell proliferation, while YAP is predominantly required for 3D body formation. Thus, it is important to use a variety of model organisms to further dissect their cellular functions.

Previous reports showed that disruption of the Hippo pathway and YAP activation induced tissue overgrowth and carcinogenesis in mice and humans [26]. In our study, we revealed a tumor suppressive function of YAP through YAP-activated cell elimination. Thus, YAP functions as both an oncogene and anti-oncogene depending on the surrounding environment.

We revealed that YAP induces ARHGAP18 GAP to form 3D organogenesis in medaka. Recently, it was reported that YAP-induced ARHGAP29 promotes metastasis in a human gastric cancer cell line [58]. And we found that YAP induces Ect2 and Fgd3 GEF to promote cell migration in mouse liver [37]. Thus, YAP regulates cell tension, epithelial-mesenchymal transition, and cell migration through cytoskeleton remodeling by Rho family GAPs and GEFs.

In this review, we described YAP function in three different contexts: (a) 3D organ formation through cell tension using medaka; (b) the role of YAP in apical extrusion using MDCK cells, and (c) the connection between YAP activation and cell elimination in damaged mouse liver. Cell tension is essential for normal development and individual organ formation. Apical extrusion and damaged hepatocyte elimination are essential for organ quality control and homeostasis. Disruption of these processes causes an increased risk of cancer. All of these cellular processes are regulated by YAP and the transcription factor TEAD, however, target gene expression is different. The molecular mechanisms underlying the expression of different YAP-target genes using the same transcription factors but in different contexts remain to be uncovered by future studies.
